# Science tikkun: a bioscience pandemic framework in a Hebrew tradition of global repair

**DOI:** 10.1186/s10020-025-01244-z

**Published:** 2025-05-16

**Authors:** Peter Hotez

**Affiliations:** 1 Department of Pediatrics and Molecular Virology and Microbiology, Texas Children’s Hospital Center for Vaccine Development, National School of Tropical Medicine, Baylor College of Medicine, Houston, TX 77030 USA; 2 Department of Biology, Baylor University, Waco, TX 76798 USA; 3 James A Baker III Institute of Public Policy, Rice University, Houston, TX 77005 USA

**Keywords:** Pandemics, Climate change, Climate health, Urbanization, Anti-science, Antisemitism

## Abstract

Over the past decade we have seen a steady increase in dangerous pandemic threats. They include two major Ebola epidemics and cholera in Africa; dengue, Zika, yellow fever in the Americas; a COVID-19 pandemic; and H5N1 in Texas. This is happening because of a confluence of modern forces including urbanization, deforestation, and climate change. Yet as pandemics emerge on a crowded and warming planet, anti-science disinformation and antisemitism impede our response. Science tikkun is an overarching framework for repair and redress. It honors the legacy of Maimonides, Teilhard de Chardin, and others who have sought reconciliation between science and religion.

## Introduction

Writing on the inherent tensions between science and religion, the 20th Century Jesuit Priest and paleontologist, Pierre Teilhard de Chardin, called for reconciliation in his breakthrough treatise, *The Phenomenon of Man* (Teilhard de Chardin 1959):

After close on two centuries of passionate struggles, neither science nor faith has succeeded in discrediting its adversary. On the contrary, it becomes obvious that neither can develop normally without the other.

Teilhard de Chardin devoted his life to the study of human evolution, but his words and actions have relevance across the biomedicine disciplines, including efforts to develop new vaccines for global infections (Hotez 2024a). Such sentiments also align with the ancient Hebrew concept of *tikkun olam* - repairing the world - to address a steady (and accelerating) drumbeat of pandemics and other global infection threats, further complicated by worsening health disinformation and political opposition to a public health response. Through a new *science tikkun* framework, religious beliefs might become allies in fighting disease and conquering anti-science sentiments. It might also resonate with a new United States (U.S.) Administration committed to faith-based approaches to problem solving.

Our current situation is becoming dire: Over the past 10 years we have seen a steady increase in the severity and frequency of dangerous pandemic threats (Hotez 2021a). This decade of global infections began in 2014 with the worst ever Ebola virus epidemic emerging in West Africa, where it killed more than 11,000 people in Guinea, Liberia, and Sierra Leone, and spread to seven other countries, including Dallas, Texas. An almost annual cadence followed. A Zika virus pandemic in 2015-16 that may have begun earlier in French Polynesia, entered Brazil and then multiple countries in the Latin American and Caribbean region to cause thousands of cases of microcephaly and other serious birth defects. Next, there was the largest cholera epidemic in modern times in Yemen which began in 2016 and continued for five years, followed by a second major Ebola virus outbreak in the Eastern Democratic Republic of the Congo and Uganda in 2018-20. Finally, the COVID-19 pandemic killed almost 15 million people, with most dying because they lacked access to a vaccine, or in some cases because they refused COVID immunizations.

Even after COVID-19, the epidemics or pandemics have not abated. African Mpox began in Africa and spread globally by 2023, while in 2024 dengue fever spread across the Americas. Dengue is one of multiple tropical infections, which are expected to threaten Texas and the U.S. Gulf Coast, along with Chagas disease, hookworm anemia, malaria, chikungunya, Oropouche, Zika virus infection (again), and even possibly yellow fever. And speaking of Texas, H5N1 avian influenza began to spill over into cattle in 2024 and now affects almost 1,000 dairy herds, mostly in California, with multiple instances of transmission to humans.

## Hot town, summer in the city

At least two 21st century drivers account for this regular appearance of deadly epidemics and pandemics (Hotez 2025a).

The first is rapid urbanization. Beginning in the 2010s or shortly before more people on our planet began living in urban centers than rural settings. Much of this urban expansion occurred as immigrants fled rural areas because of agricultural collapse, conflict, or simply economic opportunities. But the consequence is the creation of dozens of new megacities (of more than ten million people) mostly in the Global South - South Asia, the Middle East and Central Asia, the African Continent, and Latin America and Texas - where rural poverty converts to urban shantytowns. Rapid population expansions in the setting of extreme poverty collapse access to nutrition, quality shelter, and public health allowing infectious disease pathogens to flourish. Large cholera epidemics can be expected such as those that occurred in African cities in 2023-24 - Lagos (Nigeria), Harare (Zimbabwe), and Blantyre (Malawi); or in Sanaa (Yemen) in the Middle East starting in 2016. The *Aedes aegypti* mosquito that transmits dengue, chikungunya, Zika, and yellow fever also thrives in poor urban environments, such that the megacity of Sao Paulo (Brazil) and other South American cities are experiencing unprecedented dengue outbreaks (Hotez and LaBeaud 2023).

**Fig. 1:**
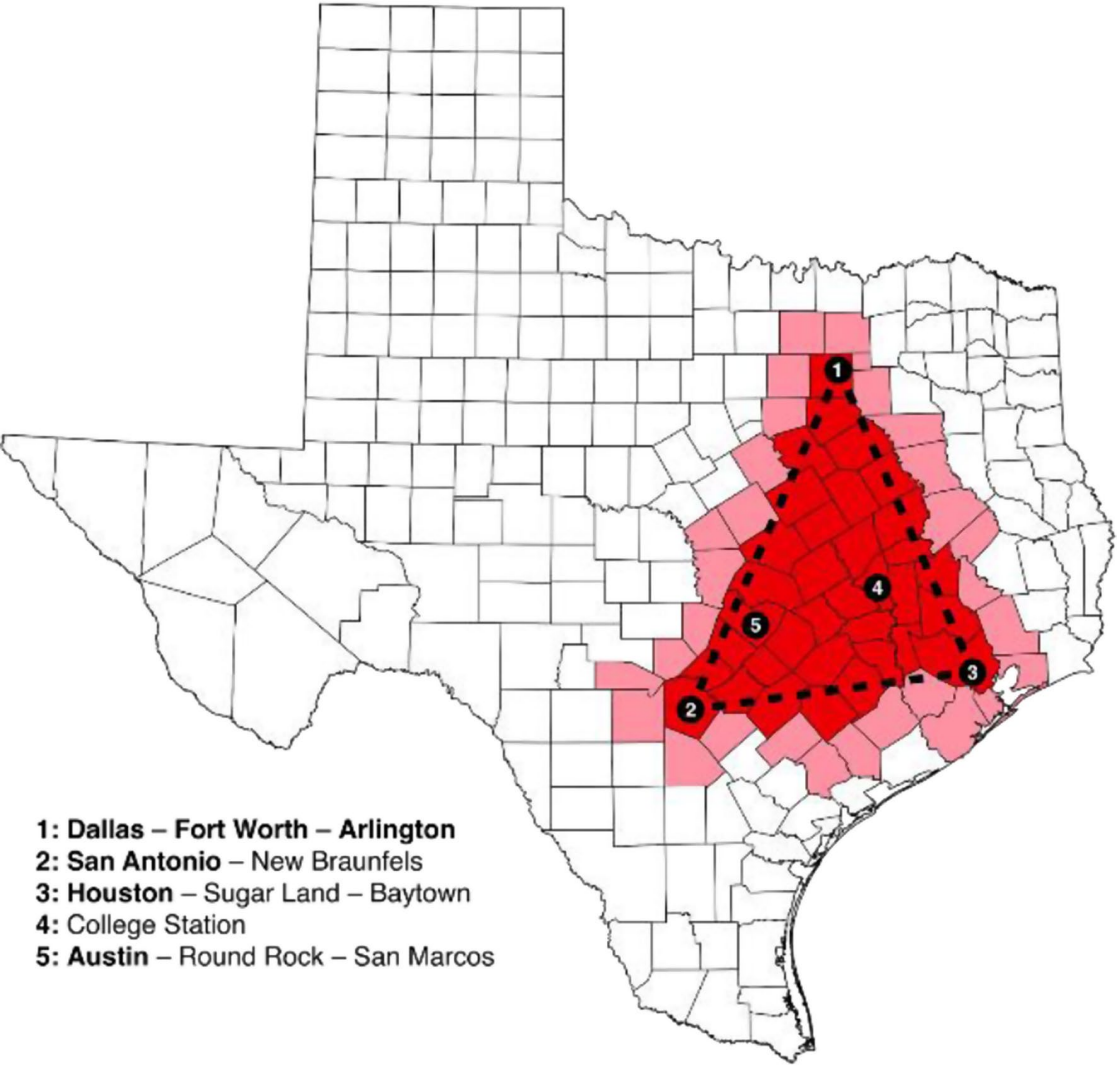
The cities and counties in or near the Texas Triangle, a megaregion of the U.S. state of Texas: City names in bold in the map legend are in the top 10 most populous Texas cities, https://en.wikipedia.org/wiki/Texas_Triangle#/media/File:Texas_Triangle,_Cities_and_Counties_map.svg

Converging with urbanization is climate change resulting in hotter temperatures, altered rainfall patterns and catastrophic weather events (Hotez 2025a). The year 2024 was the hottest on record and this past decade may account for some of our planet’s hottest years. The University of Pennsylvania climate scientist, Michael Mann, was among the first to describe the “hockey stick” curve, with the blade pointing up to reflect this recent and steep rise in global temperatures. Our urban centers are baking. The Los Angeles fires of January 2025 were the costliest in American history, while the cities of the “Texas triangle”– Austin-San Antonio, Dallas-Fort Worth, and Houston -suffer from prolonged summer heat that extends into November. In 2017, Hurricane Harvey dumped 50 inches of rain on Houston, the wettest storm on record (see Fig. 1)

These NextGen urban ovens create conditions that favor tropical infections such as cholera, arbovirus infections, and parasitic infections, as well as catastrophic respiratory virus pandemics. Climate change causes bats to seek new habitats, bringing with them their natural pathogens– coronaviruses and filoviruses (Ebola and Marburg)– closer to where humans live (Hotez 2021a). In turn, urbanization (and deforestation) brings the people closer to the bats to increase the likelihood of animal to human virus transmission leading to zoonotic spillovers and outbreaks. Similar forces may account for the increasing number of H5N1 avian influenza infections in the U.S.

## “Plandemics” and antisemitism

A new generation of pandemics and global infections is accelerating, but a hidden enabler lurks in the form of a toxic ecosystem of disinformation that prevents us from mounting a meaningful public health response. This began with false claims linking vaccines to autism during the early 2000s, despite efforts to provide evidence showing there was no connection, and the discovery of autism genes and their role in fetal neurodevelopment (Hotez 2018). Unfortunately, antivaccine activism continued to accelerate in the U.S., especially in conservative states such as Texas because of a new push in partisan political “health freedom” rhetoric. Parents requested non-medical vaccine exemptions for their children, and the number of unvaccinated schoolchildren in Texas soared (Hotez 2016).

Such antivaccine sentiments soon extended to adult immunizations during the COVID-19 pandemic. Almost one-half of the 100,000 Texans who died from COVID-19 occurred after vaccines were made widely available. They died from vaccine hesitancy or refusal (Hotez 2022). Later, the conspiracy websites and podcasters alleged that the COVID-19 pandemic was orchestrated by an elite group of globalist financiers who operated from the World Economic Forum or other organizations linked to international business. Their claim was a selected group of scientists had orchestrated the COVID-19 “plandemic” for purposes of global control (Lee et al. 2023). The plandemic rhetoric amplified after H5N1 avian influenza was discovered in Texas dairy cattle in 2024 (Cooper et al. 2025). Despite the reality that human cases of H5N1 were first detected in the late 1990s, and this current 2.3.4.4b clade began accelerating among wild birds, poultry, and dairy cattle herds in 2021, the conspiracy websites had scientists, including the author unleashing this and other viruses to disrupt the incoming U.S. Administration.

The conspiracies took on an even darker dimension when they asserted it was Jewish financiers and scientists who were responsible. The Anti-defamation League (ADL) reported an increase in antisemitic incidents in the U.S. since 2017, but these accelerated with COVID-19 pandemic. Almost 3,700 antisemitic incidents were reported in the U.S. in 2022 (U.S. Congress 2023-24). Online and on podcasts across America and globally, Jewish or Israeli scientists were blamed for creating the COVID-19 virus or for spreading the virus to profit from vaccines. As the “trifecta” of being a Jewish-vaccine-scientist, the author received an onslaught of threats online, or through emails and calls to my office, and even worse (Hotez 2023a).

It turns out that such antisemitic tropes are not new: Going back to the time of the black death in the 1300s, the Jewish people in Europe were hunted or killed in pogroms after accusations they poisoned the wells to start the plague (Hotez 2023b). In Weimar Germany during the 1920s and 30s, Jewish scientists were accused of practicing their own special brand of science and ultimately, they were forced out of German universities or required to flee to Turkey, England, or to North and South America. Einstein’s theory of relativity was dismissed as “Jewish science” or “fraud,” while Sigmund Freud and his theories of psychoanalysis were similarly attacked.

**Fig. 2:**
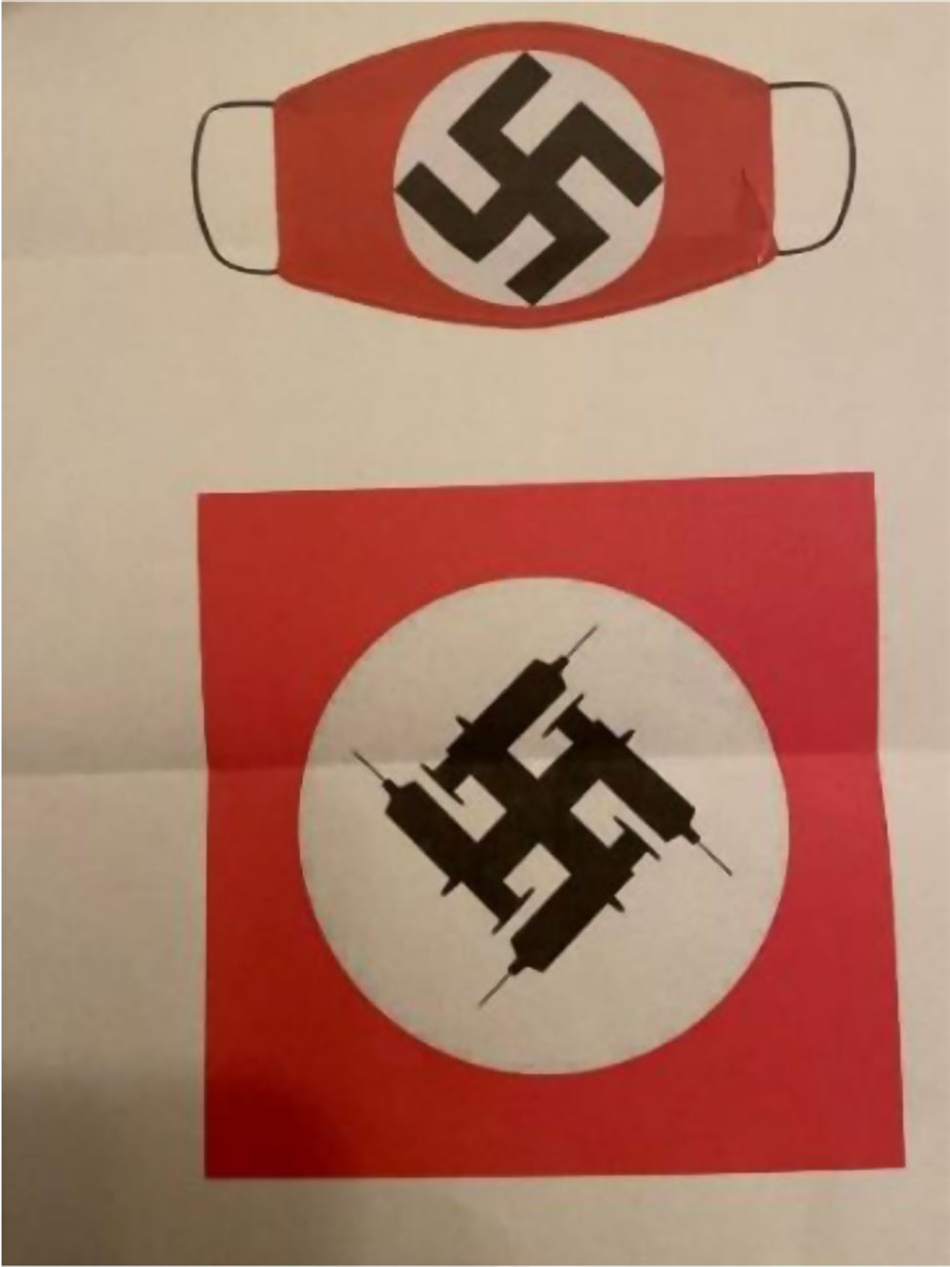
Swastikas mailed to the author’s home on two different occasions during the COVID pandemic

What goes around, comes around, and now antisemitic threats and incidents are recurring and rising in the U.S. and globally. ADL Global 100, a new report released in January 2025, revealed some alarming trends. Almost one-half of adults worldwide currently hold antisemitic attitudes, while 40% of young people believe that Jews are responsible for most world wars, and 20% of people globally have not heard of the Holocaust (Anti-Defamation League 2025). Too often, the attacks on scientists in America are accompanied by use of Nazi imagery. Antivaccine activists compare vaccines to the Holocaust, or they seek to intimidate Jewish scientists by claiming we will be executed by hanging after a military-tribunal known as “Nuremberg2” (Hotez 2023a; 2023b). Swastikas (in the shape of syringes or masks) were mailed to the author’s home on two different occasions (Fig. 2), and the author has endured in-person confrontations of people stalking his home or as an invited speaker at a Houston synagogue over the Jewish high holidays in 2021.

It is all too easy to become demoralized. The prospect of cities beset by pandemics and conflagrations can be overwhelming, while Jewish scientists are demonized as cartoon villain scientists embedded in a conspiratorial Jewish cabal profiting somehow from illness. It’s tough stuff.

## Repair

Seeking hope and repair we can look to the 20th century Jewish neurologist and psychiatrist, Dr. Viktor Frankl, who survived the Holocaust - three years in various concentration camps– and yet found resilience to not only survive but also go on to write the landmark book, *Man’s Search for Meaning* (Frankl 1959). But there is a much older concept, an ancient Hebrew tenet of *tikkun olam* or global repair (Hotez 2019; 2021b). Some scholars attribute the origins of tikkun olam to ancient Hebrew religious texts or to Maimonides (Moses ben Maimon, 1138–1204), the great Jewish physician who traveled extensively and became renowned while working in Egypt. However, a better-known expression of tikkun olam comes from Rabbi Isaac Luria, a Jewish mystic and Kabbalist, who was born in 1534 in Jerusalem but spent most of his life in Ottoman-occupied areas of the Middle East. Rabbi Luria wrote about reconnecting or repairing the world and cosmos through good works and great deeds (Hotez 2017).


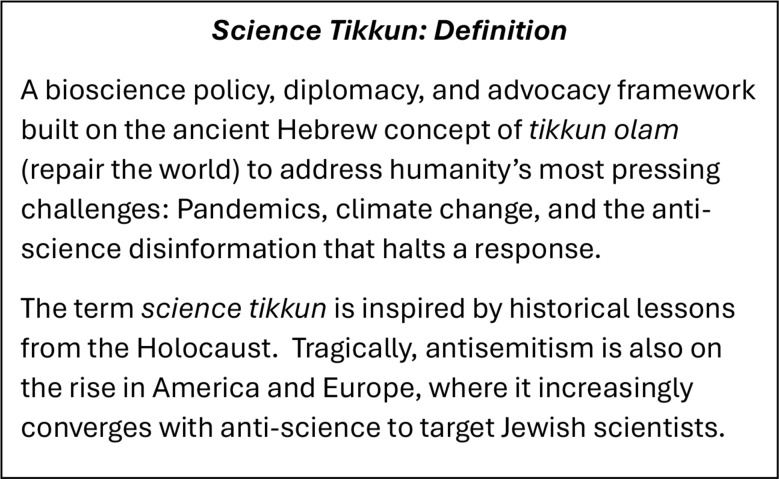


Tikkun (repair) is also applicable to modern science. The author’s original definition around “repair and redemption through science” focused mostly on science diplomacy and international scientific cooperation, citing the examples of joint U.S.–Soviet cooperation to develop and deploy vaccines for smallpox and polio for purposes of disease eradication (Hotez 2017; 2019; 2021b). But now, science tikkun can extend to a bioscience policy, diplomacy, and advocacy framework to address humanity’s most pressing challenges, and inspired by historical lessons from the Holocaust.

## Call to action

For the U.S. the fundamental tenets of science tikkun embrace an awareness of how a regular sequence pandemic threats, such as COVID-19 and zoonotic influenza infections, could become a new normal. Building new countermeasures and better public health infrastructure should be prioritized for reasons of economic and homeland security. The same might be said for the new tropical infections we can expect will emerge in the Southern U.S. (Mann and Hotez 2025).

To address the current and future rise of these infections it is feasible to implement an early warning system for climate health, in which we both expand wastewater testing for new and emerging pathogens but also conduct an innovative metagenomics approach. By sequencing the entire genome of individual mosquitoes or other arthropod vectors, together with viruses, it is possible to detect new or previously unknown pathogens affecting a community (Pan et al. 2024). This is in contrast with conventional methods using PCR primers of already known or established organisms. The metagenomics approach to unravel the full “virosphere” of a region could detect new zoonotic spillover events and provide detailed information about the virus and vector ecology of the region. In Texas and the Gulf Coast, a new virosphere project will soon be underway (Hotez 2024b).


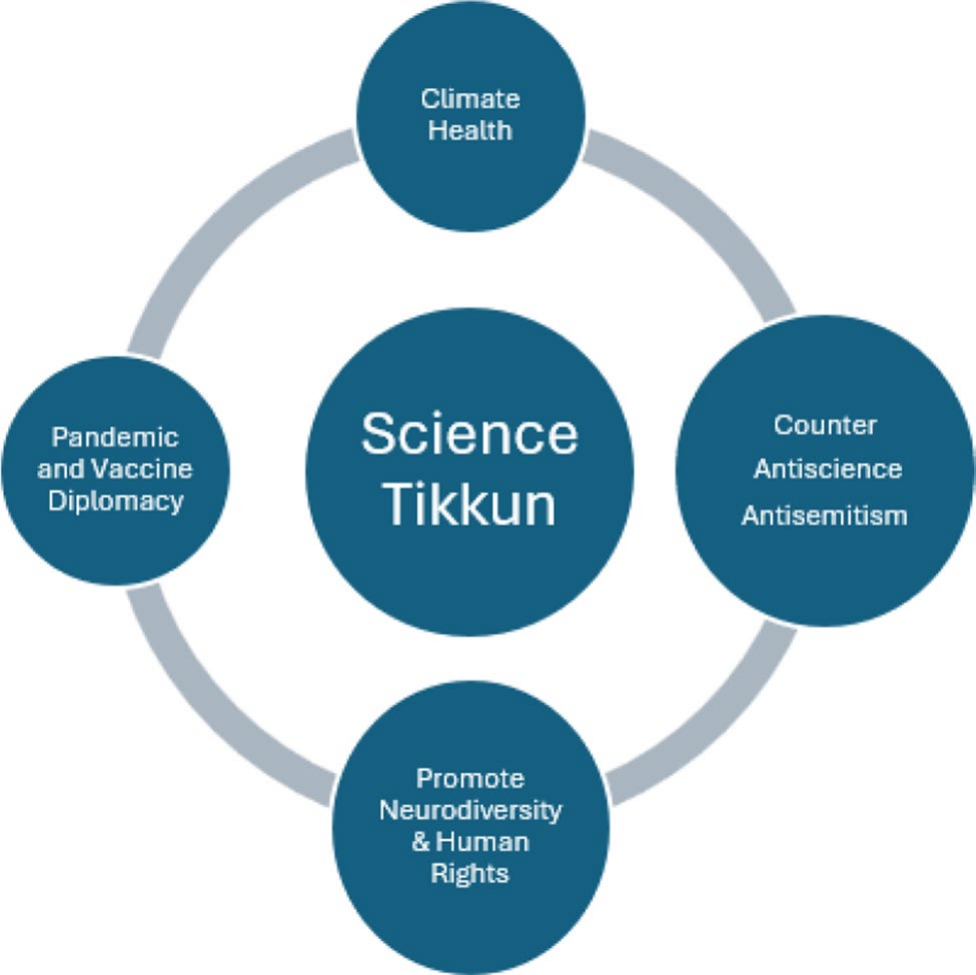


Aside from the zoonotic spillover, there is the very real situation that COVID-19 antivaccine activism has begun spilling over to childhood immunizations. Cases and outbreaks of pertussis and measles have increased by several-fold over the past year, and we could soon see widespread childhood illness across low-immunization areas, such as the 2025 measles epidemic in West Texas and adjoining areas of New Mexico and Oklahoma. In a worst-case scenario other illness, including polio, might return. Therefore, a second component of the science tikkun framework requires us to address the rise of health disinformation now deceiving parents into not vaccinating their kids. The author has outlined a counterplan to restore confidence in the vaccine ecosystem (Hotez 2025b).

Science tikkun is an overarching framework to repair the world. It relies on promoting climate health, preventing future pandemics while pursuing vaccine development between nations, explaining the science of neurodiversity, and countering expanding anti-science activism, and its fellow traveler– virulent antisemitism. It offers a connected philosophical framework for some of America’s most pressing pandemic and related challenges. It is one that honors the legacy of Maimonides, Teilhard de Chardin, and others who have sought reconciliation between science and religion over many centuries. Science tikkun might also resonate with the close ties the new U.S. Administration aspires to promote with evangelical Christians, Catholics, and other faiths. It is a new beginning.

## Data Availability

No datasets were generated or analysed during the current study.
